# Davis’ Medical History

**Published:** 1850-11

**Authors:** N. S. Davis

**Affiliations:** Prof. of Physiology and Pathology in Rush Medical College


THE NORTH-WESTERN
MEDICAL AND SURGICAL JOURNAL.

VOL. III.] NOVEMBER, 1850. [No. 4.

### Part I.—Original Communications.

### CHAPTER III.—CONTINUED.

*History of the Medical Profession from the first settlement of the British Colonies in America, to the year* 1850.—By N. S. DAVIS, M. D., Prof. of Physiology and Pathology in Rush Medical College.

[Copy Right Secured.]

ined before admission, by practitioners, themselves, without the intervention of any other class. In the following year 1807, an act was passed, making some further provisions for the internal organization of the State Society, and also prescribing a penalty of five dollars per month for practising without being authorized according to the act of the previous year. This penalty, however, was not to apply to persons using for the benefit of the sick any roots or herbs the growth of the United States. In May, 1812, the Legislature increased the foregoing penalty to twenty-five dollars for each offence, and required that all licenses in future should be deposited in the county clerk's office. In 1813, these several acts were revised and consolidated into one statute, and continued without alteration until 1818, when the Legislature passed an act increasing the term of study to four years.—But one year might be deducted, if the student had pursued classical studies that length of time after the age of sixteen years; or had attended a complete course of lectures delivered by each of the professors, on all the branches of medical science, in the medical colleges of this State or elsewhere.

In the following year another act was passed prohibiting the medical colleges from granting the degree of Doctor of Medicine to any students who had not fully complied with the requisitions of the act of 1818. The next law of importance enacted in this State was that passed by the Legislature in 1827. This leaves the term of study and the conditions for obtaining a license to practice essentially the same as before, but the twelfth section provided that "no person shall receive from the regents of the University a diploma conerring the Degree of Doctor ot Medicine unless he shall have pursued the study of medical science for at least three years, after the age of sixteen, with some physician or surgeon duly authorized by law to practice his profession, and shall also have attended two complete courses of all the lectures delivered in an incorporated medical college, and have attended the last of such courses in the college, by which he shall be recommended for his degree." And section 20th declares, that no person under the age of twenty-one years, can be entitled to practice physic and surgery in this State. Another provision of this law required all regularly licensed physicians to file a copy of their license or diploma in the county clerk's office, and become members of the County Society in the county of their residence, before they were entitled to collect pay for their services.

Such are the essential features of the legislative enactments, adopted from time to time for the internal organization and regulation of the medical profession in the State of New York; and though exceedingly defective in many respects, they exerted a decided and beneficial influence over the great mass of the profession. The frequent contact with each other, and the mutual interchange of sentiments, which took place in the County Societies, soon led the practitioners to a more thorough knowledge of each other, and, consequently, to the adoption of by-laws and rules of ethics for their mutual government. This again led to a far more dignified and honorable intercourse with each other in private practice. The meetings of these Societies were occupied in the reading of essays, the relation of cases, and the discussion of topics connected with medical practice, and generally with the diseases of their own counties; and not few of the papers read at their meetings would do credit to any learned body. The Presidents of the Societies were also generally required to deliver an address at each anniversary meeting. Some of these were published either in pamphlet form or in the medical journals—some found their way into the transactions of the State Society, and all were not only interesting, but well calculated to excite a spirit of investigation, and divert the attention of practitioners from the petty jealousies of private competition to the study of medicine as a science.

In the early part of this period, the number of medical colleges in the Union were but few, and the degrees conferred by them much less sought after than at present; and hence many of the candidates for admission into the ranks of the profession were examined and licensed by the County Societies, and the fees derived from the granting of *such licenses were in most cases devoted to the purchase of books to constitute a county library. By this means, the latest and best medical works were constantly being brought within the reach of every practitioner; and hence these libraries became powerful auxiliaries in the general diffusion of medical knowledge. The same ends were stil farther promoted by the action of the State Society, which was organized February 3d, 1807. Thus we find the Society at its first meeting directing each member "to present a geological and topographical description of the county in which he might practice, and

*For a brief history of this Society, including the names of its officers from year to year, see the "U. S. Medical and Surgical Journal, vol. ii.; and also the published transactions.

also a history of such diseases as might prevail in his particular place of residence, etc." Accordingly, in the following year, we find reports in compliance with this direction, from no less than seven members, viz:—Dr. Alex. Sheldon, of Montgomery; Dr. David R. Arnell, of Orange; Dr. Wm. Wheeler, of Dutchess; Dr. John Stearns, of Saratoga; Dr. Hugh Henderson, of Jefferson; Dr. Horatio Powell, of Clinton, and Dr. Lyman Cook, of Westchester. At this second annual meeting (Feb., 1808), the Society presented to the profession still stronger inducements to engage in medical investigations, by offering three premiums, viz.:—a medal of the value of fifty dollars for the "best dissertation on the topography, geology, and mineralogy of any county in the State, together with the prevalent diseases in said county." Another of the value of twenty-five dollars for the second best dissertation on the same subject; and a third premium, consisting of a medal of the value of twenty-five dollars, for "the best dissertation on the causes, and best method of preventing and curing the typhus mitior, or low bilious fever, which prevailed in different counties of the State."

These offers called out several well written essays, and the first premium on the topography, etc., was awarded to Dr. John Stearns, of Saratoga county. The efforts to improve medical science, and elevate the character of the profession, thus early and actively commenced, have been continued with unremitting zeal by this Society until the present time. But though the influence of the State and County Societies has been highly beneficial to the profession, yet that influence has been in some measure counteracted by defects in the laws, and other circumstances, over which they had no control. Thus, while the law of 1812 increased the penalty for practicing without a license or diploma to tweniy-five dollars for each offense, it was rendered almost entirely void in practice, by the proviso, "that it should not be so construed as to prevent any person from prescribing for the benefit of the sick, any roots, barks, or herbs, the growth and produce of the U.S."

Although the design of this provision was, undoubtedly, simply to shield nurses in their common practice of using simple domestic teas or infusious, in cases of sickness not considered sufficiently severe to require a physician, yet in practice it was made to cover every species of empiricism, it being only necessary to plead the use of indigenous remedies. Hence, although the statutes of New York have contained, apparently, strict prohibitory or protective laws in regard to medical practice for more than thirty years, those laws have been practically inoperative from their own defects, and cannot, therefore, be considered any practical test of the practical bility or impracticability of suppressing quackery by penal enactments. Another important defect in the laws regulating ting the education of the profession consisted in the entire omission of any standard of preliminary education as a reqnisite, before commencing the study of medicine. This defect not only existed in the laws regulating the profession, but equally so in the rules adopted by medical societies and colleges for granting the diploma ; hence we have been, and still are, annually witnessing the ridiculous spectacle of young men, possessing the high and dignified title of Doctor of Medicine, conferred by instutions called colleges and universities, who are destitute of even a competent knowledge of English grammar. These facts sufficiently explain why, notwithstanding the existence of a good internal organization, and the united efforts of State and County Societies during a period of forty years, every species of qnackery still abounds in this State—they show, too, that the repeal of that part of the law prescribing a penalty of twenty-five dollars for practicing without a license or diploma, and also, that which renders the unlicensed incompetent to enforce payment for their services, which took place in 1844, was rather the repeal of an obsolete form than the removal of an operative law.

The medical laws of New Jersey were so amended in

1816 as to prohibit all unlicensed persons, who were not already engaged in practice, from entering on those duties in that State, under a penalty of twenty-five dollars for each offense. Such persons were also disqualified from collecting any compensation for medical services. But, instead of containing the neutralizing proviso which we have noticed in the laws of New York, it declared that, "this Act shall be so construed as to prevent all irregular bred pretenders to the healing art, under the names or titles of practical botanists, root or Indian doctors, or any other name or title, involving quackery of any species, from practicing their deceptions, and imposing on the ignorance and credulity of their fellow-citizens." Some unimportant alterations in the medical laws of this State were made by the Legislature in the years 1818, '23, '25, '30, '38; but the main features still remain in full force. And though the penal provisions against unlicensed practititioners are very seldom enforced; yet the influence of their State, and District or County Societies, has been most salutary in promoting friendly intercourse, stimulating investigation, and elevating the professional character. State and district medical Societies were organized at an early period in all the New England States. We have, in a previous chapter, mentioned the origin of those in Massachusetts, Connecticut, and New Hampshire. The Maine Medical Society was incorporated in 1821, and that of Rhode Island, in 1812. The regulations adopted in all these States were very similar. They all required the establishment of State or District Boards of Censors, for examining and licensing candidates to practice; also some degree of preliminary education, a term of medical study not less than three years, and the attainment of the age of 21 years. In Massachusets, Rhode Island, and New Hampshire, the Boards of Censors were unconnected with the medical colleges of those States; and the laws required all persons intending to commence practice, whether educated in those States or already licensed by the institutions of other States, to apply to some one of the Boards of Censors for a license before they were authorized to enforce payment for their services. In Connecticut and Maine, but one Board of Censors was established in each State, which was authorized to examine all candidates, whether for a license or the higher degree of M. D. These Boards are composed, in the one State of the Medical Faculty of Yale College, associated with an equal number of Censors appointed by the President and fellows of the Connecticut Medical Society, the President of the Society always being one of the number: and in the other, of the Medical Faculty of Bowdoin College, and an equal number of Censors chosen by the Maine Medical Society.

In January, 1822, the Medical Society of the State of Deleware was authorized, by an act of the Legislature, to appoint a Medical Board of Examiners, consisting of fifteen members, whose term of office was to continue five years; and who were directed to examine and license all candidates for admission into the profession in that State. The requisites for admission to an examination by such Board were, three year's study with some respectable practioner, the attendance on one full course of lectures in some medical college, and the attainment of twenty-one years of age. But graduates of respectable medical colleges were licensed on the exhibition of their diplomas, without an examination. The same penalties were enacted against unlicensed practitioners as in the State of Maryland. The Medical Society of the District of Columbia was incorporated by an act of Congress in 1819, with power to appoint a Board of Examiners, composed of five practitioners, whose duties and privileges were the same as those appointed by the Delaware Medical Society. And the same penalty was enacted against unlicensed and irregular practitioners. The States of S. Carolina, Georgia, Alabama, Mississippi, and Louisiana have all had laws of a similar character, for the regulation of medical education and practice.

In 1817, the Legislature of South Carolina enacted a law establishing two medical boards of examiners, one in Charleston and the other in Columbia. They were required to examine all applicants for permission to practice in that State, except such as had received a diploma from some medical college, and grant licenses to those they deemed qualified. And every one practicing without such license was liable to be indicted and fined in a sum not exceeding five hundred dollars, and imprisoned a term not exceeding two months. These regulations continued in force until 1838, when all restrictions and penalties were abolished by an act of the State Legislature. The act by which a fine of five hundred dollars was imposed on all who should practice physic in Georgia without a license from the board of physicians, was passed by the State Legislature in 1826, and continued in force until 1835, when it was repealed. In 1839, the examining board of physicians was re-organized, and again invested with power to examine applicants and grant licenses ; but with the following proviso, which nullifies the whole act, viz.: "Provided nothing in the said revised act be so construed as to operate against the Thompsonian or Botanic practice, or any other practitioner of medicine in this State." It should also be mentioned that the Thompsonians have had for several years a regularly incorporated college in that State, with all the usual collegiate powers; but at present its existence appears to be merely nominal.

In Alabama, an act was passed in December, 1823, requiring the establishment of five boards of medical examiners in the State, each consisting of three members, elected by a joint vote of both houses of the State Legislature. Their powers and duties in regard to examining and licensing candidates were the same as those existing in South Carolina. The penalty for practicing without such license or a diploma from some medical college, was a sum not exceeding five hundred dollars for each offense. But the examining boards were all abolished some eight or ten years since, which operated as a repeal of all law on the subject.

The medical laws of the state of Mississippi are coeval with the existence of the State itself. They provided for the establishment of three boards of examiners, appointed by the State Legislature. These boards were required to examine all candidates for permission to practice medicine and surgery in theil respective districts, whether graduates of a medical college or not, and grant licenses to such as they found qualified. By an act passed in 1827, all licenses were required to be filed in the clerk's office of the county where the holder of said license should commence practice, within six months from the time of said commencement. All attempts to practice without procuring a license from one of the examining boards, and having it duly recorded in the clerk's office, was punishable by a fine not exceeding five hundred dollars, and imprisonment not exceeding six months. It was further made the duty of the county clerk to present a complete list of all licenses recorded in his office to the grand jury of each county court; and such grand jury were required to present to the court all such persons as, from their own knowledge, or from information given by others, were practicing physic or surgery without a license. In 1829, another act was passed by the Legislature, authorizing the establishment of a "Medical Society of the State of Mississippi." These laws were very complete, and effectually accomplished the object for which they were designed. But when the State Constitution was revised in 1834, the several boards of examiners weie omitted, which operated as a repeal of all restraints on the practice of medicine in that State; and though the State Medical Society has maintained its organization, yet, since the year 1834, there have been no legal provisions for discouraging quackery in any of its forms. The first laws relating to the practice of physic and surgery in Louisiana were passed in 1808. They were revised and amended in 1816--17--20.

In the latter year, two medical boards were established, one for each supreme judicial district in the State. These boards were composed of six members each, appointed by the governor, with the advice and consent of the Senate, with one apothecary attached to the board in the first district. These boards were to examine all applicants for license to practice in their respective districts, and license such as were found qualified; but such as had graduated at a respectable medical college were permitted to obtain a license on exhibiting their diploma, without an examination. The apothecary attached to the board in the first district, was to examine and license apothecaries, who were under the same regulations as practicing physicians. The penalties prescribed for violating the laws of this State, by practicing without a license, were a fine of one hundred dollars for the first offense; and for the second, a fine not exceeding two hundred dollars, and imprisonment not more than one year. The attorney-general was required to prosecute for all violations of the laws. The licenses were required to be filed in the parish or county-clerk's office. the same as in Mississippi. A State Medical Society was incorporated in Tennessee, in 1830, wiih a board of censors authorized to examine and license all persons who may present themselves for examination, touching their skill in the practice of medicine and surgery. No term of study, or other preliminary condition, is required of the applicant, except that he be twenty-one years of age, and of good moral character; and no penalties are provided against practicing without a license.

The Legislatures of Ohio, Indiana, and Michigan, have all passed laws incorporating State and County or District Medical Societies, with power to appoint censors, and license candidates to practice much the same as in New York. These laws also laid some moderate restrictions on unlicensed and irregular practitioners; but, as in most of the older states, all these restrictions have been repealed within the last ten years, leaving the regularly organized societies to maintain the honor of the profession, and protect the interest of the community as best they could.

From the hasty glance we have now taken of medical legislation throughout the Union, it will be observed that during the first quarter of the nineteenth century, the legislatures of all the older states, except Pennsylvania, Virginia and North Carolina, enacted laws for the avowed purpose of protecting the citizens against the impositions of ignorance and empiricism, and of promoting medical science. That these were the real motives for enacting laws on this subject, and especially the first one named, that of protecting the citizens against imposition, is abundantly shown by the preambles and titles attached to the several acts themselves.

The idea of protecting the profession, or investing it with special privileges, seems to have been the discovery of a later period, as we shall see in the sequal. Again, the business of examining and licensing candidates for admission into the ranks of the profession, was not only uniformly committed to medical men, but, with very few exceptions, those men were also selected by the profession, or rather by regularly organized medical societies. Another important fact is, that during this period, medical societies were regularly organized in almost every state in the Union; hence the same effects that we have ascribed to the medical organization of New York, was felt to a greater or less extent throughout the whole Union. The intercourse between medical men was everywhere more dignified, medical intelligence was more rapidly and generally diffused, the importance of a good knowledge of anatomy, physiology, and chemistry, became better appreciated, and in the same proportion, medical colleges were more generally patronized, and medical literature cultivated.

Such were the legitimate and highly beneficial influences exerted by medical associations throughout the country. The number of graduates from medical colleges during the earlier part of this period was comparatively small, and in some of the states even those were obliged to procure licenses before entering into practice—far the larger share of those who entered the regular profession being examined and licensed by the boards of censors, appointed by state or district Medical Societies—a considerable fund was thus derived, which, in most instances, was appropriated, as in New York, to the support of the several societies, and the promotion of their legitimate objects;---holding thus, as it were, the keys of the profession, and aided, to some extent, by the funds derived from licenses, the medical organization of the several states and districts was actively and vigorously sustained.

The organization of Medical Societies throughout the Union also brought into beneficial action another powerful stimulant to human enterprise--viz., ambition. Every society, whether county, district, or state, must have its official stations, its posts of honor; and hence, every right-minded member of these societies would be so influenced in his conduct as to gain the esteem and confidence of his professional brethren, without which he could not hope to be honored by them. But while the internal organization of the profession was thus rapidly improving its character and influence, other agencies were brought into operation, some of which exerted a widely different effect, both on the profession and the community.

At the commencement of the period now under consideration, only about five hundred students were in attendance on the lectures in the four or five medical colleges then existing in the Union, and the whole number of graduates for the year 1807 did not exceed fifty. No sooner, however, did the study of anatomy, physiology, chemistry, etc., become better appreciated through the influences already detailed, than the students attending the colleges began rapidly to increase, and the number of colleges increased also. The small degree of prosperity which attended the Medical Department of Columbia College, led many members of the profession to use their influence to establish another college in that city. Accordingly the regents of the University of New York granted a charter for a new college in 1807, called the "College of Physicians and Surgeons of New York." This school was placed under the direction of a board of trustees, consisting of the whole medical society of the city and county of New York, and the degree of M. D. was conferred by the regents of the University of the State, on the recommendation of the trustees and faculty of the college. The first course of lectures was given in the winter of 1807--8, to a class of fifty-three students, and was continued regularly thereafter until the present time.

In 1810, the Medical Department of Columbia College was finally discontinued, leaving the College of Physicians and Surgeons the only one in the State, with a class of students numbering eighty-two. But instead of that rapid prosperity which the friends of the institution, and the regents of the University now anticipated, the very numerous board of trustees, being mostly medical practitioners in the immediate vicinity of the college, soon became distracted by opposing councils, and jealousies between them and the members of the faculty, which caused much difficulty, and greatly retarded both the prosperity of the college, and the progress of medicine, in that city.

The Medical Department of the University of Maryland, in Baltimore, was incorporated by the Legislature of that State in 1807, and was soon supplied with an able faculty, and has continued to enjoy a fair share of public confidence and prosperity until the present time.

In 1810, a medical department was attached to Yale College, at New Haven, but the first course of lectures was not delivered until the winter of 1813--14, since which time they have been regularly continued, the class usually numbering between fifty and one hundred, and the number of graduates from three to twenty-nine annually,

The next medical institution established in the country was at Fairfield, Herkimer County, New York, in 1812, called the "College of Physicians and Surgeons of the Western District." It was chartered by the regents of the State, with the same powers and duties as the College of Physicians and Surgeons of the city of New York. The degrees were conferred by the regents on the recommendation of the faculty and trustees of the college. The first course of lectures was given in the winter of 1813--14, to a class of thirty-three students. The course of this institution was marked by a uniform degree of prosperity until 1834, when the class numbered two hundred and seventeen. From this period it began gradually to decline, owing to the influence of neighboring schools, and in 1840 the whole faculty resigned their places, and no successors were appointed.

Two new colleges were established in 1818, one at Castleton, Vermont, called the "Vermont Academy of Medicine," and the other at Lexington, Kentucky, called the "Medical School of Transylvania University." The degrees of Castleton Medical College were conferred by Middlebury College until 1828, since which time they have been conferred by the college under its independent charter. The number of students in attendance have varied from twenty-four, in 1818, to one hundred and thirty, in 1836, and the number of graduates averaged about twenty-five annually. Since 1835, two courses of lectures have been given annually; one in the spring and another in the fall. The Transylvania Medical School rapidly attained a high degree of prosperity, the number of students averaging over two hundred annually, and the graduates varying from seven, in 1820, to eighty-three, in 1835.

The Medical College of Ohio was incorporated in 1819, located at Cincinnati, and has continued with a fair share of prosperity until the present time.

In the following year, the Medical School of Maine was established at Brunswick, in connection with Bowdoin College. The first course of lectures was delivered in 1821, to a class of twenty-one students, while in 1836 the number had increased to one hundred, and the number of graduates to twenty-seven. The medical school attached to Brown University, at Providence, Rhode Island, was established in 1821, but was discontinued after a few years. In the year following, the Medical School of the University of Vermont was commenced at Burlington, but was also discontinued previous to 1840. The Berkshire Medical School was established at Pittsfield, Massachusetts, in 1823, and has continued its annual courses of lectures to classes varying from seventy-three to one hundred and seventeen, until the present time.

The next medical college established in the country was at Charleston, South Carolina, in 1824, called the "Medical College of South Carolina." This school appears to have been under the control of the Medical Society of the State, and enjoyed a fair share of public patronage until dissensions arose between the faculty and the governing body, which caused the former to resign their places in 1832. Their places were immediately filled, and the annual courses of instruction continued, but to a greatly reduced class for several years. In the meantime, the professors who had resigned obtained from the State Legislature, in 1833, a charter for another school in the same city, called the "Medical College of the State of South Carolina." The first class attending the new college in the winter of 1833--4, numbered one hundred and three, and the school seems to have sustained a fair degree of prosperity up to the present period. During the year 1824, another school of medicine was also established in Philadelphia, called the "Jefferson Medical College." But the first course of lectures was given in the winter of 1825--6, to a class of one hundred and ten students. It has since acquired a degree of popularity, second only to that of the University of Pennsylvania, so long established in the same city.

In 1825, two other colleges were established, viz.—the Medical School of Columbian College, in the District of Columbia, and the Medical School of the University of Virginia, at Charlottsville. The organization of this latter school is somewhat peculiar. Its periods of instruction continue through ten months of each year, and all the branches are taught by three professors, in much the same manner as other sciences are taught in collegiate institutions. The number of medical students in attendance, in 1835--6, was sixty-three. The Washington Medical College was established at Baltimore, Maryland, in 1827, its degrees being conferred by Washington College, in Pennsylvania until 1833, when it obtained a regular charter from the Legislature of Maryland. In 1834, the number of graduates was only ten, and in 1838--9, the whole number of students was fifty-three.

The next medical institution was the "Medical College of Georgia," located at Augusta, and incorporated in 1830. The first course of lectures, however, was not given until the winter of 1832--3, when twenty-seven students were in attendance.

In 1834, the Willoughby University, at Willoughby, Ohio, was incorporated, and supplied with a medical faculty, who gave their first course of lectures in the winter of 1835--6 to a class of twenty-three students. It enjoyed a moderate degree of prosperity until the year 1847, when the medical department was transferred to Columbus, and re-organized under the name of the "Starling Medical College," in honor of Lyne Starling, who made the very liberal donation of thirty thousand dollars for the benefit of the institution, and five thousand dollars more for the establishment of an hospital.

During the year 1835, no less than four medical schools were added to the number already established in the Union, viz.—the Medical College of Louisiana, at New Orleans; the Medical Institution of Geneva College, at Geneva, New York; the Medical Department of Cincinnati College, at Cincinnati, Ohio; and the Vermont Medical School at Woodstock, Vermont. The Louisville Medical Institute, at Louisville, Kentucky, and the Medical Faculty of the University of the City of New York, were established in 1837, and the Medical Department of Hampden Sidney College, at Richmond, Virginia, in 1838. In the following year, still two more were added, viz.—the Albany Medical College at Albany, New York, and the Medical Department of Pennsylvania College, at Philadelphia.

During the period intervening between 1840 and the present time (1850), no less than thirteen new medical colleges have been established, viz.—two at St. Louis, Missouri, called the Uuiversity of Missouri, and the St. Louis University; one at Chicago, Illinois, called the Rush Medical College; one at Cleveland, Ohio, called the Western Reserve College; one in Indiana, called the Indiana Medical College, located at La Porte; two in Philadelphia, called the Philadelphia College of Medicine, and the Franklin Medical College; one at Buffalo, New York, called the Medical Department of the University of Buffalo; one at Memphis, Tennessee, called the Memphis Medical College; one at Evansville, and another at Indianapolis, in Indiana; one at Davenport, Iowa, called the College of Physicians and Surgeons of the Upper Mississippi; and one in Michigan, located at Ann Arbor, being a department of the University of that State.

Of the forty-three medical colleges which have thus been organized, we believe that seven have been wholly discontinued, while others have merely changed their names or localities, leaving thirty-six now in active operation in the United States. Of these, seven are in the eastern or New England States, nine in the middle, seven in the southern, and thirteen in the western. It will be noticed that only six medical colleges were organized prior to 1810, five between 1810 and 1820, eight between 1820 and 1830, eleven between 1830 and 1840, and thirteen between 1840 and 1850. The number of students in attendance on the several colleges, at each period of ten years, may be stated as follows, together with the number of graduates, viz,:

| WHOLE NUMBER OF STUDENTS. | | GRADUATES. | |
|---|---|---|---|
| In 1810 | 650 | In 1810 | 100 |
| " 1820 | 964 | " 1820 | 181 |
| " 1830 | 2125 | " 1830 | 597 |
| " 1840 | 2800 | " 1840 | 775 |
| " 1850 | 4500 | " 1850 | 1300 |

These numbers are not claimed as entirely accurate, owing to the difficulty of obtaining complete and reliable information on the subject; but they are sufficiently so for all the purposes of comparison. They illustrate very clearly what we have already stated when detailing the organization of medical societies, viz., that the medical colleges were patronized just in proportion as the importance of the fundamental branches of medical science became better and more universally appreciated, through the medium of such societies. Thus, during the ten years following 1820, a period when the medical societies of most of the states were in their most active and influential state, the number of students attending the medical schools were more than doubled, and the number of graduates increased threefold. It is a fact also worthy of notice, that the number of graduates has been constantly increasing faster than the whole number of students. Thus, in 1810, the ratio of graduates to the whole number of students attending the schools, was 1 to 6, 5; in 1820, 1 to 5, 3; in 1830, 1 to 3, 5; in 1840, 1 to 3, 6; in 1850, 1 to 3, 4.

But this exceedingly rapid increase in the number of students who resort to the medical colleges, and the number taking degrees, by no means indicate an equally rapid increase in the whole number of those pursuing the study of medicine. For, it must be remembered, that medical examiners had been appointed in a large majority of the states in connection with their social organization; and, during the first quarter of the present century, a much larger number of students were probably examined and admitted by these numerous boards of examiners than by the colleges. But a large majority of the states having made the college diploma a legal admission into the profession, with all its rights and privileges, it soon became the paramount object of the student's pursuit. This, together with the absence of all preliminary requisites in regard to general education, and the many facilities afforded by the rapid multiplication, and consequent competition of the colleges with each other, caused the licenses from local examining boards to be comparatively neglected. Thus, in 1820, only thirty-eight students received the degree of M. D. from the medical colleges of the State of New York, while three times that number were examined and licensed by the censors of the state and county societies. In 1830, the graduates from the medical colleges of the same state numbered fifty-six, those licensed by the censors of the state society, seventeen, and probably one hundred more by the numerous county boards. While, in 1846, the whole number of graduates in the state was two hundred and forty-six, the number licensed by the censors of the state society was only three, and those reported by the county societies, five. Although it is quite probable that a few were licensed by the county societies who were not reported to the state society, yet the whole number for two or three years previous had not averaged ten annually. The effects of this rapid change were twofold, The local societies being gradually deprived of the funds derived from granting licenses, soon found their libraries neglected, and their regular meetings diminishing both in interest and in the numbers in attendance. This gave rise to a general feeling of indifference on the part of the profession, during the prevalence of which many local societies ceased to maintain an active existence; and whatever laws for restraining quackery had been enacted by the legislatures of the several states, were, with very few exceptions, repealed. While, on the other hand, the great increase of patronage bestowed on the medical schools, literally begot a mania for college making. And this mania was rendered still more intense by the regulations almost universally adopted as requisites both for graduation and license. These gave no credit for any courses of instruction, however extensive and complete, except such as should be delivered in a regularly-established college. Hence, every professional man who became ambitious of distinction as a teacher, sought a professorship in some college as the only position in which that ambition could be gratified. And as there are always more such men than there are places for them to fill, the constant and inevitable tendency is to the creation of more places. If the State Legislature, to whom application is made for an act of incorporation, happens to be so stupid as not to perceive the necessity of establishing a new school, a bargain is soon struck with some literary college, already possessing the right to confer degrees, to furnish the necessary diplomas, and straightway a new medical college, with all the usual honors and privileges, springs into existence. To so great an extent has this spirit been carried, that scarcely a single year has passed since 1830, without witnessing the birth of one or more of these institutions. If the standard of preliminary education had been elevated, and the requisites for graduation increased in proportion to the multiplication of schools, no evils would have resulted to the profession or the community; but, there being practically no standard of preliminary education, and the professors, with only two or three exceptions, being the sole judges of the student's qualifications, the addition of every new college only served to increase the competition, and add to the facilities for obtaining diplomas. Indeed, to such an extent has this spirit and practice been carried, that any young man can obtain the once high and honorable title of M. D. for a less expenditure of time and labor than it takes to obtain the primary literary title of A. B.

It is true that the professors in many of the colleges would gladly have stayed this tendency of things; but it was not in their power. Hence, with only two or three recent exceptions, eight months' attendance on a medical college—three years' study, including the eight months—twenty-one years of age—and a thesis on some medical subject, constitute, practically, all the qualifications required of the candidate for medical honors, previous to his examination.

If we contrast these with the qualifications required in other countries, or with those originally required by the founders of the old school in Philadelphia, we shall cease to be surprised either at the crowded state of the profession, or at the heterogenous mixture of all grades, characters, and degrees of attainment which it presents. The impossibility of any faculty of five or six professors doing anything like justice to the whole field of medical science in the short space of four months to say nothing of the perfect absurdity of attempting to crowd any amount of material so vast and varied on the mind of the student in so short a space of time, early attracted the attention of many enlightened members of the profession, among whom were some of the ablest professors connected with our schools. Hence, attempts were made from time to time to extend the courses of college instruction, and elevate the standard of medical attainments; but so rapid was the increase of new schools, and so active the rivalship, that every attempt to produce concert of action among the schools of even a limited number of states proved abortive. We believe the earliest of these efforts were made by the colleges of New England, some of whom, in good faith, carried into practice an agreement to extend their courses of instruction; but others, wholly neglecting the movement, soon induced all to return to the former limits.

In 1835, the faculty connected with the Medical College of Georgia called the attention of their professional brethren to the same subject; and, both by correspondence and through the columns of the *Southern Medical and Surgical Journal*, urged the propriety of a national convention of delegates from all the colleges, not only to agree upon a longer term of instruction, but also upon a higher standard of medical education. These efforts were favorably responded to by a large proportion of the colleges then existing, but the time and place for holding the convention was left to the medical faculty of the University of Pennsylvania, who, by declining to

[See page 501.]

